# Evidence of SARS-CoV-2 infection in companion animals from owners who tested positive for COVID-19 in the Valley of Mexico

**DOI:** 10.1007/s11033-023-09099-5

**Published:** 2024-01-25

**Authors:** Edith A. Fernández-Figueroa, Deborah V. Espinosa-Martínez, Haydee Miranda-Ortiz, Erika Ruiz-García, Juan M. Figueroa-Esquivel, Miriam L. Becerril-Moctezuma, Anallely Muñoz-Rivas, César A. Ríos-Muñoz

**Affiliations:** 1 Núcleo B de Innovación en Medicina de Precisión, Instituto Nacional de Medicina Genómica, Mexico City, Mexico; 2 Posgrado en Ciencias del Mar y Limnología, Universidad Nacional Autónoma de México, Mexico City, Mexico; 3 Laboratorio de Arqueozoología, Subdirección de Laboratorios y Apoyo Académico, Instituto Nacional de Antropología e Historia, Mexico City, Mexico; 4 Unidad de Secuenciación, Instituto Nacional de Medicina Genómica, Mexico City, Mexico; 5 Laboratorio de Medicina Traslacional, Instituto Nacional de Cancerología, Mexico City, Mexico; 6 Universo Animal, Hospital Veterinario, Texcoco, State of Mexico Mexico; 7 Laboratorio de Diagnóstico Genómico, Instituto Nacional de Medicina Genómica, Mexico City, Mexico; 8 Facultad de Ciencias, Universidad Nacional Autónoma de México, Mexico City, Mexico

**Keywords:** Human-animal transmission, Nucleocapsid, RT-qPCR, SARS-CoV-2

## Abstract

**Background:**

Little is known about the companion animals which tested positive in Mexico for Severe Acute Respiratory Syndrome Coronavirus 2 (SARS-CoV-2) infection. Due to this, it is that we have documented the infection of companion animals, via an exploratory approach in two localities of the Valley of Mexico, in which the companion animal owners tested positive for COVID-19.

**Methods:**

Oropharyngeal and nasopharyngeal swabs were collected from 21 companion animals. Also, a Reverse-Transcription Quantitative Polymerase Chain Reaction was used to test five probes in three SARS-CoV-2 genes. More than one-third (5/14) of these samples were positive for SARS CoV-2 corresponding to dogs.

**Results:**

This research translates into the first available report on companion animals with SARS-CoV-2 infection in the most populated area of Mexico. Samples were added chronologically to previous reports prepared in other areas of the country, from February through November 2022.

**Conclusion:**

Although SARS-CoV-2 infection in dogs is not as common as in other animals, our results suggest that it can be transmitted to dogs by their owners to a greater extent than previously reported.

**Supplementary Information:**

The online version contains supplementary material available at 10.1007/s11033-023-09099-5.

## Introduction

Severe Acute Respiratory Syndrome Coronavirus 2 (SARS-CoV-2) has accounted for the COVID-19 disease, which was declared as a global pandemic by the World Health Organization on March 11th, 2020 [1]. The first imported cases in Mexico occurred in February 2020 and community transmission occurred in March 2020, when the Public Health Emergency of International Concern was established [2]. As of November 11, 2023, Mexico has accumulated 7,712,840 cases with a rate of cumulative incidence of 5,877.37 cases per 100,000 inhabitants (information updated to November 11th, 2023) [3].

Given the close contact between humans and companion animals, and considering the high number of cases worldwide, research on reverse transmission of SARS-CoV-2 from humans to other mammals has been carried out [4–6]. While it has been reported that ferrets and cats are more susceptible to SARS-CoV-2 infection [7], cases of dogs have also been reported, thus suggesting human-animal transmission [8]. Previous surveillance efforts have been made in Chile [9], Mexico [10, 11], Colombia [12, 13] and Brazil [14], and have identified four cases of SARS-CoV-2 infection in dogs in Colombia [12, 13] and eight cases in Brazil [14]. Mexico reported that a total of 43 (of 135) dogs and cats had tested seropositive for SARS-CoV-2, via the ELISA test (representing with this a 32% of sampled companion animals in the city of Puebla) [11], whilst there were no cases reported in 130 samples collected from 30 cats and 100 dogs from the state of Veracruz [10]. The Valley of Mexico comprises Mexico City and its metropolitan area (of which the State of Mexico is part) and according to estimations, there are 5,495,746 companion animals solely in Mexico City [15]. Still, there is no information available of SARS-CoV-2 cases in companion animals, where almost more than two million of human cases of COVID-19 cases have been reported [3].

## Materials and methods

Nasal samples were taken from 21 companion animals, two cats and 19 dogs. In geographical terms, eight dogs from Mexico City were sampled, as well as two cats and 11 dogs of the State of Mexico. The samples were taken by veterinarians from two collaborating veterinary clinics; one of these located in Xochimilco (Mexico City) and the second, in Texcoco (State of Mexico). This sampling was conducted within the period of February to November 2022 during regular visits to both clinics. Owner consent was obtained for purposes of the present research, though approval from an Ethics Committee was not required. Eligibility criteria for companion animal enrollment included that all owners tested positive for COVID-19 by Reverse-Transcription Quantitative Polymerase Chain Reaction (RT-qPCR), and that the owners had been diagnosed at least two days prior the sampling of their companion animals. The owners remained in contact with the personnel of the veterinary clinics and none of them had severe COVID-19 nor were hospitalized. All owners declared be very close of the companion animals including sleep together and sharing the same food with it. Given that the companion animal owners only agreed to the swabs, oropharyngeal and nasopharyngeal samples were taken and preserved in 1 mL of TRIzol.

Samples with 1 mL of TRIzol were homogenized for 2 min in a vortex, incubated during 10 min at room temperature (RT), upon extraction of RNA. TRIzol solution was taken and mixed with 300 μL cold chloroform (Sigma). This solution was incubated at RT for 5 min and then centrifuged at 19,357×*g* for 12 min at RT. The aqueous phase (400 μL) was recovered and 700 μL of isopropanol (Sigma) at RT were added. The resulting solution was mixed for 15 s and incubated during 10 min at RT. Then, the solution was centrifuged at 19,357×*g* for 12 min at RT. The supernatant was discarded and 1 mL ethanol 80% (Sigma) was added; the solution was mixed for 20 s and centrifuged at 19,357×*g* during 12 min at RT. The ethanol was disregarded, the excess was dried at RT and the pellet was suspended in 50 μL RNAse free water and stored at -20 °C until use.

Total RNA was analyzed for detecting SARS-CoV-2 with the aid of two different kits, and both RT-qPCR were performed via the 7500 FAST Real-Time PCR System. For the first kit, N1 and N2 probes (nucleocapsid protein gene) were included through the GoTaq Probe 1-Step RT-qPCR System (Cat. A6121, Promega) in a reaction volume of 20 μL containing: 10 μL GoTaq, 1.5 μL probe (N1, N2 or RNAse P), 0.4 μL GoScript, 3.1 μL Nuclease-Free water and 5 μL RNA. As positive control, Synthetic DNA Gene N of SARS-CoV-2 (Cat. 10006625. 2019-nCoV_N_ Positive Control, IDT) was used. The thermal profile was performed, as follows: 45 °C during 15 min, 45 °C during 15 min, 95 °C during 2 min, 40 cycles at 95 °C for 15 s and 60 °C for 1 min. Cycle threshold (Ct) values were assessed by qRT-PCR described in the CDC 2019 Novel Coronavirus (2019-nCoV) RT-PCR Diagnostic Panel instructions (CDC-006-00019).

The second kit used for viral RNA detection was the TaqMan 2019-nCoV Assay Kit v1 (Cat. A47532, Applied Biosystems) targeting SARS-CoV-2 Orf-1, S and N genes. Using TaqMan Fast Virus 1-Step Master Mix (Cat. 4444434, Applied Biosystems) in a reaction volume of 20 μL containing: 5 μL Master Mix, 1 μL probe (Orf-1, S, N) and 1 μL probe of RNAse P, 8 μL Nuclease-Free water and 5 μL RNA. TaqMan 2019-nCoV Control Kit v1 (Cat. 47,533, Applied Biosystems) was used as a positive control. The thermal profile went, as follows: 25 °C during 2 min, 50 °C during 15 min, 95 °C during 2 min, 45 cycles at 95 °C for 3 s, and 60 °C for 30 s. Samples were considered positive when at least two of the three targets were amplified with Ct values < 38 as per the manual. RNAse P was used as internal control in both experiments [16], and only those samples which amplified in the RT-qPCR real-time endogenous gene (Ct ≤ 36), were considered as valuable for analysis, in terms of detecting the SARS-CoV-2 virus.

It is important emphasize that the algorithms for molecular detection of SARS-CoV-2 in humans were also applied in this study for the companion animal sample testing [17]. Taking oropharyngeal samples does not guarantee an effective collection of material; for this reason, we included a negative extraction control (NEC) during the extraction to avoid cross-contamination [17]. Additionally, the quality of the nucleic acids extraction was analyzed via an endogenous gene that indicates that there is sufficient genetic material in the sample. Then, viral detection was conducted along with the analysis for molecular diagnosis.

## Results and discussion

Owners of companion animals while attending a regular veterinary visit, were invited to participate in the study. The only inclusion criterion was that companion animal owners had been diagnosed with COVID-19 disease prior to the sample collections from the 19 dogs and two cats. Five dogs were given a medical examination, whereas the other 16 companion animals from owners attending a wellness visit to the veterinary clinic, were voluntary donors (Table 1). According to results (in which N1, N2, Orf-1, S and N probes were used), five dogs were positive for SARS-CoV-2 using, via the use of Promega and Thermo Fisher kits (Table 1, Supplementary information S1). For the first time, study results showed evidence of positive infection in companion animals in Mexico City, and the State of Mexico. This is through the use of gold standards for viral detection.

**Table 1:** Characteristics of companion animals tested positive to RNA after the third and during the fourth wave of COVID-19 human cases from February 2022 to November 2022 in the Valley of Mexico

Companion animal	Breed	Reason to attend at hospital	Age (years)	City	SARS-CoV-2 detection (qPCR)	*RNAseP	*N1	*N2	*Orf-1	*S	*N
Female dog	Mixed breed	Polydipsia, ascites	10	Texcoco	Positive	29	39	37	37	34	38
Female dog	Bichon	Sample donor (Asymptomatic)	15	Mexico City	Positive	31	38.5	Negative	38	37	37
Female dog	Maltese	Sample donor (Asymptomatic)	ND^**+**^	Mexico City	Positive	19	Negative	38	36	36	38
Female dog	Belgian Malinois	Sample donor (Asymptomatic)	4	Mexico City	Positive	36	39	37	38	36	36
Female dog	Siberian Husky	Breathing problems and gastric bleeding	ND^**+**^	Texcoco	Positive	30	35	37	Negative	34	38
Female dog	Mixed breed	Head trauma	1.5	Texcoco	Negative	33	Negative	Negative	Negative	Negative	Negative
Female dog	Mixed breed	Breathing problems	0.9	Texcoco	Negative	31	Negative	Negative	Negative	Negative	Negative
Female dog	Australian Shepherd	Sample donor (Asymptomatic)	ND^**+**^	Texcoco	Negative	34	Negative	Negative	Negative	Negative	Negative
Female dog	Mixed breed	Sample donor (Asymptomatic)	ND^**+**^	Texcoco	Negative	33	Negative	Negative	Negative	Negative	Negative
Female dog	Mixed breed	Sample donor (Asymptomatic)	17	Texcoco	Negative	34	Negative	Negative	Negative	Negative	Negative
Female dog	Collie	Sample donor (Asymptomatic)	0.5	Texcoco	Negative	30	Negative	Negative	Negative	Negative	Negative
Female dog	Mixed breed	Sample donor (Asymptomatic)	1.5	Texcoco	Negative	34	Negative	Negative	Negative	Negative	Negative
Female dog	Mixed breed	Sample donor (Asymptomatic)	ND^**+**^	Mexico City	Negative	34	Negative	Negative	Negative	Negative	Negative
Female dog	Mixed breed	Sample donor (Asymptomatic)	ND^**+**^	Mexico City	Negative	35	Negative	Negative	Negative	Negative	Negative
Female dog	Pitbull	Nasal bleeding	0.8	Mexico City	Invalid	X	X	X	X	X	X
Female dog	Poodle	Sample donor (Asymptomatic)	5	Mexico City	Invalid	X	X	X	X	X	X
Female dog	Andalusian Bodeguero	Sample donor (Asymptomatic)	17	Mexico City	Invalid	X	X	X	X	X	X
Female dog	Poodle	Sample donor (Asymptomatic)	12	Texcoco	Invalid	X	X	X	X	X	X
Female dog	Poodle	Sample donor (Asymptomatic)	10	Texcoco	Invalid	X	X	X	X	X	X
Female cat	Domestic European	Sample donor (Asymptomatic)	12	Texcoco	Invalid	X	X	X	X	X	X
Male cat	Domestic European	Sample donor (Asymptomatic)	0.5	Texcoco	Invalid	X	X	X	X	X	X

*X* invalid

*Number in the box represent the Ct obtained in the qPCR

^**+**^Correspond to not determined ages of the companion animals

The samples were taken in the period of decline, during the 3rd wave (February 2022) until the end of the 4th wave (November 2022) in Mexico [3], whereas previous studies have sampled during earlier waves, from October–December 2020 [10] and November 2020–March 2021 [11]. Clinicopathological findings have been reported regarding the American bully [12], German shepherd dog [13], Yorkshire terrier, poodle, and mixed breed dogs [14]. Nonetheless, dogs can also be infected with SARS-CoV-2, but remain asymptomatic [12], as some of those reported during this study. In one of the previous reports prepared for Mexico, companion animals that were not direct in contact with the owners, tested negative for COVID-19 (being the foregoing, the possible cause of not having found infected animals in the past) [10]. Thus, the eligibility criteria included a close relationship with the companion animal owners, as previously stated. The likelihood of transmission is high due to the close contact between owners and companion animals. On the other hand, this risk proves to be lower than that presented by cats or ferrets [7]. Despite the obtained data, still further information is needed to establish the susceptibility of dogs to SARS-CoV-2, which apparently is not common. As previously detailed, our results demonstrate infection by SARS-CoV-2, concerning its detection in companion animals that were in contact with humans who had the virus [18]. Although there have been prior [8, 9], this is the first study conducted on companion animals that tested positive for the virus in the Valley of Mexico. The same incorporates two of the states with the higher number of companion animals and in which there is a higher incidence of COVID-19 virus cases in humans within the country [3, 13].

## Conclusion

Monitoring efforts have been limited about companion animals and SARS-CoV-2 in Latin America. Despite cats and ferrets being more susceptible than dogs, the closeness of companion dogs with the infected owners raises the odds of human-companion animal transmission of SARS-CoV-2 [14]. Different clinicopathological findings have been reported in dogs, but asymptomatic cases may be dismissed, given that only one of the five positive cases presented breathing problems and gastric bleeding. The rest were asymptomatic (Table 1) and there is no knowledge of presence of symptoms, upon completion of the study. The previous surveillance efforts reported for Mexico encompass different periods of time during the pandemic of SARS-CoV-2, which include earlier waves [10, 11]. Our study includes a period of time not previously reported at the final stage of the 3rd wave (considered the most intense wave in the country) and the 4th wave, with the Omicron variant [3]. In our study, it was impossible to perform serological assays, given that owners did not allow the taking of blood samples. Serological assays would confirm the presence of neutralizing antibodies for SARS-CoV-2, in swab samples with an absence of viral RNA [11]. Also, our sampling was conducted on companion animals whose owners tested positive for SARS-CoV-2 and not randomly [10, 11] and even if owners referred to having close interaction with their companion animals, no more detailed data survey was included in our study. It is important to highlight that the number of human-positive cases of COVID-19 in the Valley of Mexico is the highest in the country [3] and that companion animal cases may be dismissed due to scarce monitoring. Companion animal cases could be underestimated given the lack of surveillance.

## Supplementary Information

Below is the link to the electronic supplementary material.

Supplementary file1 (DOCX 593 kb)

## Data Availability

All relevant data is found and incorporated in the manuscript and supplementary data.
