# Supplementary material for: Evidence of SARS-CoV-2 infection in companion animals from owners who tested positive for COVID-19 in the Valley of Mexico
Source: Mol Biol Rep. 2024 Jan 25;51(1):186. doi: 10.1007/s11033-023-09099-5 (PMC10811044; doi:10.1007/s11033-023-09099-5)

Supplementary Material 1. RT-qPCR of SARS-CoV-2 N, S and Orf-1 genes. (A) Amplification of targets N1 and N2 (nucleocapsid protein gene) of SARS-CoV-2 and RNAse P as an endogenous gene of RNA quality extraction of the companion dog analyzed. (B) Amplification of targets N1 and N2 of SARS-CoV-2 of synthetic DNA of the N gene using IDT probes (positive control). (C) Amplification of targets N, Orf-1 and S of SARS-CoV-2 and RNAse P of the companion dog using Thermo Fisher probes. (D) Amplification of targets N, Orf-1 and S of SARS-CoV-2 as positive controls of the kit (2 X 103 copies/μL) (Thermo Fisher). In all figures horizontal lines represent the cycle thresholds for each probe; above these values the samples are considered positive.


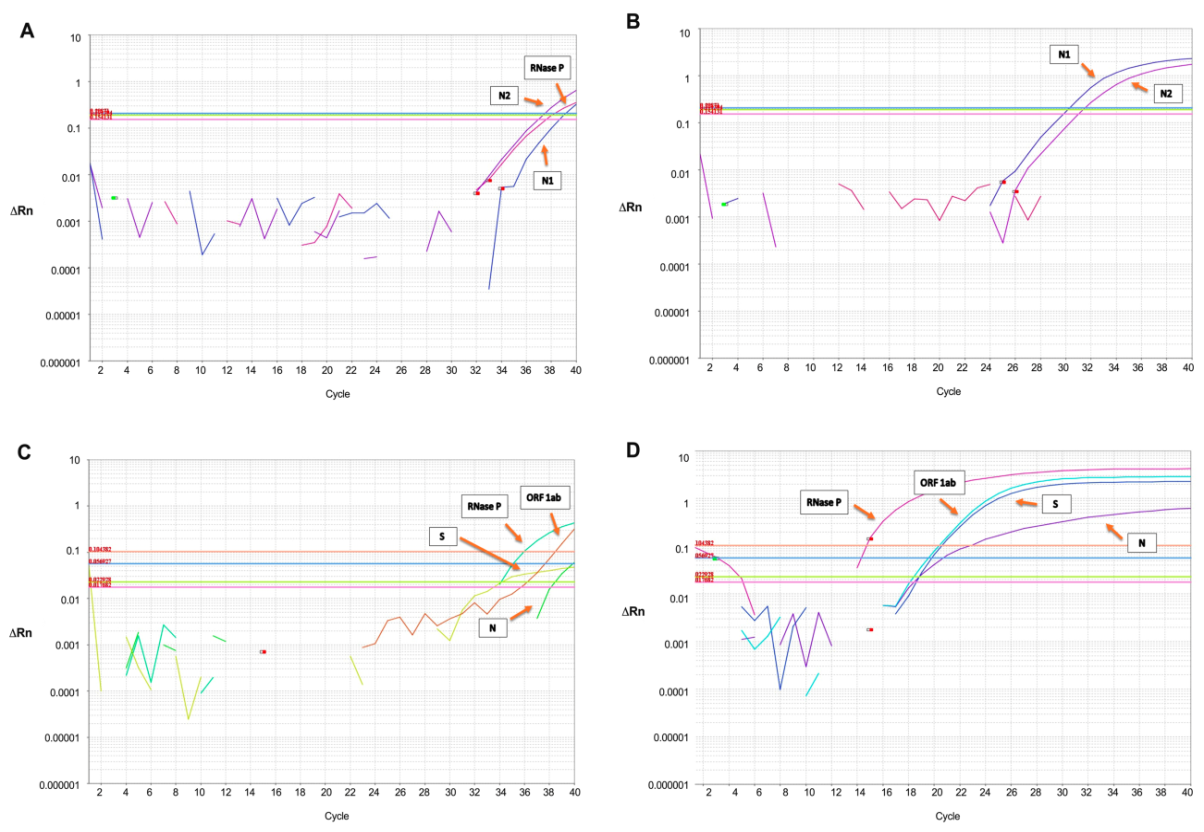

Supplement: Supplementary file 1 — Supplementary file1 (DOCX 593 kb) [file 11033_2023_9099_MOESM1_ESM.docx]
